# Volatile oil from *Saussurea lappa* exerts antitumor efficacy by inhibiting epithelial growth factor receptor tyrosine kinase-mediated signaling pathway in hepatocellular carcinoma

**DOI:** 10.18632/oncotarget.12962

**Published:** 2016-10-28

**Authors:** Xuejing Lin, Zhangxiao Peng, Xiaohui Fu, Chunying Liu, Yang Xu, Weidan Ji, Jianhui Fan, Lei Chen, Lin Fang, Yao Huang, Changqing Su

**Affiliations:** ^1^ Department of Molecular Oncology, Eastern Hepatobiliary Surgical Hospital & National Center of Liver Cancer, Second Military Medical University, Shanghai 200438, China; ^2^ Department of Biliary Tract Surgery, Eastern Hepatobiliary Surgical Hospital, Second Military Medical University, Shanghai 200438, China; ^3^ Jiangsu Center for The Collaboration and Innovation of Cancer Biotherapy, Xuzhou Medical College, Xuzhou 221002, China

**Keywords:** VOSL, hepatocellular carcinoma, epithelial growth factor receptor, signaling, xenograft model

## Abstract

Hepatocellular carcinoma (HCC) treatment remains lack of effective chemotherapeutic drugs, therefore, discovering novel anti-HCC drugs is a very attractive and urgent task. In this study, we reported VOSL (volatile oil from *Saussurea lappa* root) exhibits potent therapeutic effect on SMMC-7721 xenografts without obvious side effects. In the *in vitro* experiments, VOSL inhibited HCC cell proliferation by arresting cell cycle at S and G2/M phases, and induced HCC cell apoptosis by activating the Caspase3 pathway. VOSL also decreased the capability of HCC cell migration and invasion through MMP-9 depression. Moreover, mechanistic study indicated that VOSL can act as an epithelial growth factor receptor (EGFR) inhibitor to suppress EGFR activation and then to suppress its downstream MEK/P38 and PI3-K/Akt pathways. These results suggested that VOSL may be a novel anti-HCC drug candidate.

## INTRODUCTION

Hepatocellular carcinoma (HCC) is a most malignant cancer which generally arises from various kinds of chronic liver diseases [1–3]. The five year survival rate of HCC patients after surgery is only about 20%-30%. Frequent recurrence, metastasis and multi-drug resistance are the main reasons for the high mortality rate of HCC [4, 5]. Up to now, sorafenib is the only effective target chemotherapeutic agent for late-stage HCC patients, however, its therapeutic effect is still rather disappointing. Therefore, to discovernovel drugs is an urgent work for the treatment of HCC.

During a long time, natural products have been served as important sources of novel lead structures for discovery of anticancer agents as their diverse ‘drug-like’ structure and ‘biologically friendly’ molecular qualities [6–9]. According to a recent analysis, at least 73 clinically approved anticancer drugs are plant-derived agents, including some well-known chemotherapeutic agents, such as vinblastine, etoposide, paclitaxel, topotecan [8, 10].

The root of *Saussurea lappa*, has been used in the treatment of cancer for thousands of years in China. It includes a variety of bioactive ingredients like volatile oil, sterols, alkaloids, organic acids and so on. Our previous studies demonstrated that the volatile oil from *Saussurea lappa* root (VOSL), sesquiterpene lactones-rich fraction, contains the most important anti-cancer constituents [9]. Thereinto, costunolide (Cos) and dehydrocostuslactone (Dehy) are the main natural sesquiterpene lactones in VOSL, account for about 75% by weight. Numerous researches reported that these two compounds exhibited potential anti-cancer activities towards various types of cancer, including leukemia, bladder cancer, breast cancer, prostatic cancer, and so on [10]. Cos and Dehy have anti-hepatitis B virus activity, which is very important for prevention of liver cancer [11]. Therefore, we thought that VOSL and its main bioactive constituents, Cos and Dehy, may be potential drug candidates for prevention and treatment of HCC.

Our previous study also revealed that Cos and Dehy exhibited synergistic anti-cancer effect *in vivo*. VOSL showed stronger inhibition effects on human breast cancer MCF-7 xenografts than Cos or Dehy alone. Moreover, VOSL not only inhibited the growth of tumors, but also maintained weight and vitality of tumor-burdened nude mice, which is a great significance to “tumor-burdened survival” [12]. However, the synergistic anti-tumor mechanism of VOSL remains vague. Therefore, we investigated the anti-HCC efficiency and molecular mechanisms of VOSL in order to promote its pre-clinical research, which may offer a novel chemotherapeutic agent for HCC prevention and treatment.

## RESULTS

### VOSL suppresses HCC cell proliferation and HCC xenograft growth

**Figure 1:**
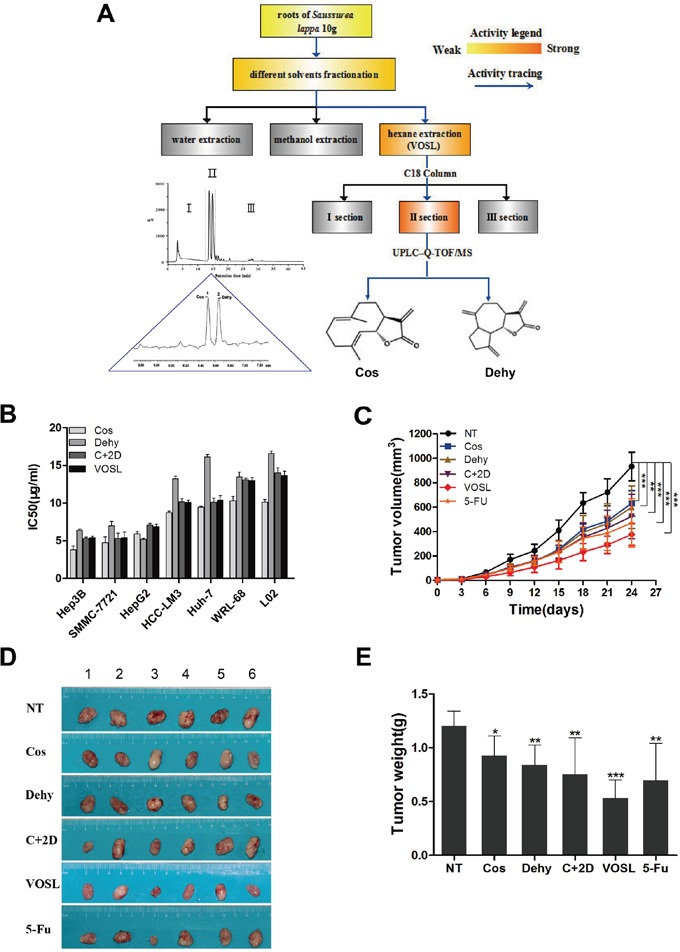
Therapeutic effects of VOSL and its main active ingredients on liver cancer **A.** A flow diagram of VOSL isolation and identification of its active ingredients using a bioactivity-oriented screening platform. **B.** Cell lines were exposed to Cos, Dehy, C+2D and VOSL for 48 h. Cell viability was measured by MTT assay and the IC50 value of the tested samples was calculated using the Trimmed Spearman-Karber Method. **C.** SMMC-7721 cells were injected subcutaneously into the axillary fossa of nude mice until the tumors reached about 2-3 mm in diameter. The mice were treated with Cos, Dehy, C+2D, VOSL and 5-FU at the indicated concentrations, n=6 in every group. Tumor volume was measured and compared; **p < 0.01 and ***p < 0.001 compared with the negative control (NT) group. **D** and **E.** The tumors were harvested and weighed after 24 times of administrations; *p < 0.05, **p < 0.01 and ***p < 0.001 compared with the NT group.

VOSL, Cos and Dehy were obtained from the dry roots of *Saussurea lappa* through a bioactivity-oriented screening platform in previous study (Figure 1A) [9]. MTT assay showed that SMMC-7721 and Hep3B cells were more sensitive to these compounds. Simultaneously, the inhibitory rate of VOSL on normal liver cells, especially WRL-68 cells, was the lowest, which revealed the relatively weak toxicity on normal cells (Figure 1B).

SMMC-7721 xenografts in nude mice were used to evaluate the anti-HCC effects of VOSL and its active ingredients, and the results demonstrated that VOSL revealed the best anti-HCC activity among five test groups (Figure 1C, 1D and 1E). The inhibitory rates of VOSL, 5-Fu, C+2D, Dehy, and Cos on SMMC-7721 xenografts are 55.71%, 42.22%, 37.51%, 30.23%, and 23.15%, respectively, after intraperitoneal injections for 24 times.

### VOSL inhibits clone formation of HCC and modulates cell cycle progression

**Figure 2:**
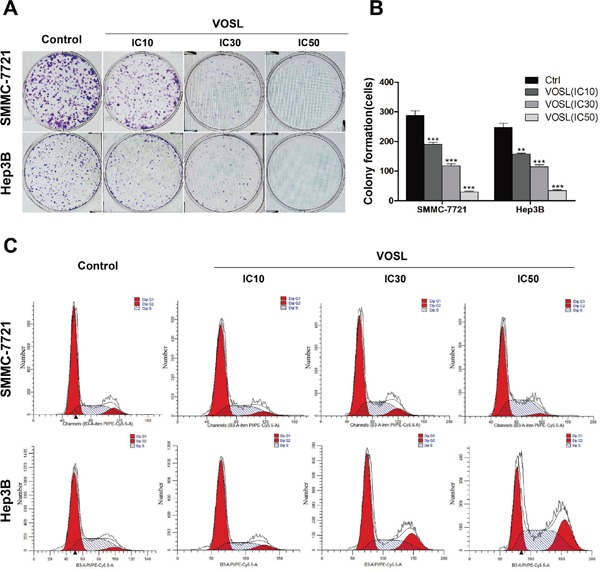
VOSL inhibits colony formation of HCC and influences the distribution of cell cycle **A** and **B.** Cells were treated with VOSL at different concentrations (IC10, IC30, IC50) for 48 h, and the colony formation was presented as mean ± SD; **p < 0.01 and ***p < 0.001 compared with the control group. **C.** HCC cells were exposed to VOSL (IC10, IC30, IC50) for 48 h and cell cycle was detected by flow cytometry (Detail data shown in Table 1).

To investigate the long-term cytotoxicity of VOSL and its active ingredients on HCC cells, cell clone formation assay was carried out on SMMC-7721 and Hep3B cells. The results revealed that VOSL, C+2D, Cos and Dehy dose-dependently inhibited the clone formation of SMMC-7721 and Hep3B cells (Figure 2A and 2B; Supplementary Figure S1). The uncontrolled cell cycle progression is one of the hallmarks of cancer, therefore, agents targeting this process may be potential chemopreventive drugs [10]. Our results demonstrated that VOSL, C+2D, Cos, and Dehy dose-dependently modulated SMMC-7721 cell cycle progression through S phase increase combined with G0/G1 depletion. Moreover, Cos changed Hep3B cell cycle progression by increasing S phase and decreasing G0/G1 and G2/M phases. However, VOSL, C+2D and Dehy changed Hep3B cell cycle progression by increasing S and G2/M phases combined with decreasing G0/G1 phase (Figure 2C and Table 1).

**Table 1:** Cell cycle distribution after treatment of Cos, Dehy, C+2D or VOSL for 48 h

Cell lines	Treatment	Concentration	G0/G1 (%)	S (%)	G2/M (%)
SMMC-7721	Control		63.22±2.62	28.57±1.97	8.21±0.67
	Cos	IC10	59.43±0.62	33.85±0.59 ^*^	6.72±1.00
		IC30	50.71±0.73 ^**^	39.44±0.60 ^**^	9.86±0.15
		IC50	41.77±0.30 ^***^	51.01±0.22 ^***^	7.22±0.33
	Dehy	IC10	62.88±0.37	30.84±0.61	6.28±0.48
		IC30	55.57±0.83 ^**^	35.89±0.66 ^*^	8.54±1.30
		IC50	49.44±0.90 ^*^	46.22±0.94 ^***^	4.34±1.18 ^***^
	C+2D	IC10	63.91±1.52	31.25±0.89	4.84±1.23 ^*^
		IC30	53.26±0.39 ^**^	37.86±0.94 ^**^	8.88±0.84
		IC50	45.62± 0.72 ^***^	48.57±0.45 ^***^	5.80±1.09 ^*^
	VOSL	IC10	62.67±1.55	29.84±0.70	7.42±0.96
		IC30	57.99±1.69 ^*^	34.09±0.29 ^**^	7.91±1.96
		IC50	54.96±1.68 ^**^	41.12±1.45 ^***^	3.92±0.46 ^*^
Hep3B	Control		67.44 ±1.35	24.45±1.18	8.11±0.30
	Cos	IC10	65.85±3.14	25.41±1.06	8.74±3.29
		IC30	59.54±1.29 ^**^	34.35±2.23 ^**^	6.11±2.82
		IC50	49.71±2.09 ^***^	46.93±1.29 ^***^	3.36±2.05^*^
	Dehy	IC10	64.47±3.62	26.14±0.86	9.40±3.25
		IC30	60.16±2.34 ^**^	36.01±2.16 ^**^	3.83±2.92
		IC50	34.83±1.74 ^***^	46.90±1.08 ^***^	18.27±1.77 ^***^
	C+2D	IC10	64.88±2.14	29.31±0.67 ^**^	5.81±2.19
		IC30	44.51±3.00 ^***^	36.22±0.95 ^***^	19.27±2.10 ^***^
		IC50	32.72±0.82 ^***^	48.73±1.15 ^***^	18.54±1.46 ^***^
	VOSL	IC10	53.43±0.74 ^***^	27.92±1.65 ^*^	18.65±1.03 ^***^
		IC30	57.56±1.68 ^**^	35.11±1.67 ^***^	7.34±1.63
		IC50	33.55±0.64 ^***^	43.78±1.25 ^***^	22.68±1.48^***^

Note:

* *p* < 0.05,

** *p* < 0.01 and

*** *p* < 0.001 compared with the corresponding control group.

### VOSL induces HCC cell apoptosis

**Figure 3:**
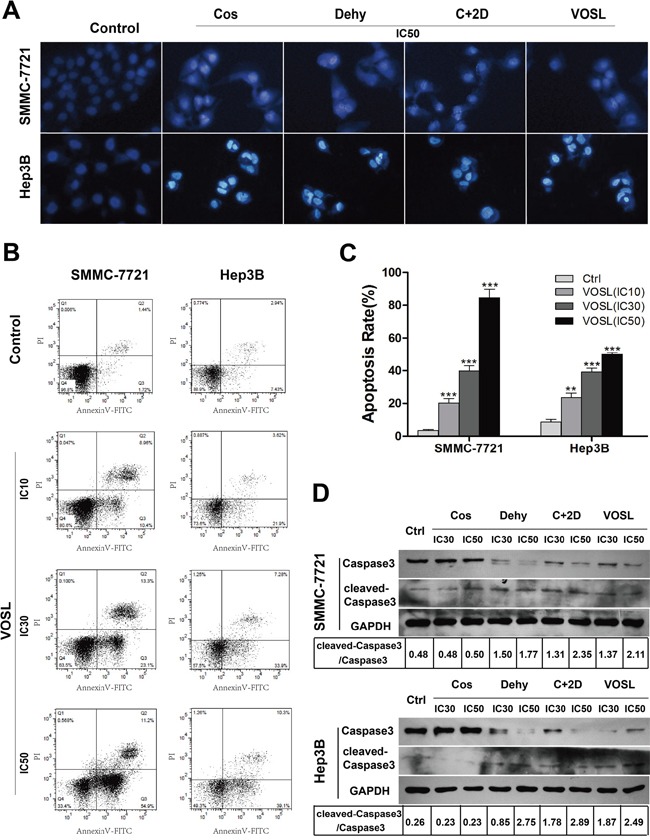
VOSL induces HCC cell apoptosis **A.** Apoptotic changes induced by IC50 of Cos, Dehy, C+2D snd VOSL treatments for 48 h were observed by Hoechst 33342 staining in HCC cells. **B** and **C.** Apoptotic cells were determined by flow cytometry. The apoptotic percentages were presented as mean ± SD; **p < 0.01 and ***p < 0.001 compared with the control group. **D.** The Caspase3 and cleaved-Caspase3 levels in HCC cells were examined by Western blot after 48 h treatment.

Hoechst analysis revealed that cell nuclei of HCC cells in the tested groups experienced a stronger blue fluorescence than those in the control group, which meant that VOSL, C+2D, Cos, and Dehy treatment can induce apoptosis of SMMC-7721 and Hep3B cells accompanied with nucleus condensation (Figure 3A).

Annexin V-FITC/PI staining showed that VOSL, C+2D, Cos, and Dehy treatments led to a dramatic increase of apoptotic ratio in both SMMC-7721 and Hep3B cells (Figure 3B and 3C; Supplementary Figure S2). Active Caspase3 is the primary markers of cells undergo apoptosis. Cleaved-Caspase3 and Caspase3 were measured by Western blot at 48 h later after treatment. The results showed VOSL, C+2D and Dehy treatments up-regulated the expression of cleaved-Caspase3 and down-regulated the expression of Caspase3 in SMMC-7721 and Hep3B cells. However, the expression of Caspase3 and cleaved-Caspase3 in the Cos-treated group had no obvious changes compared with the control group, which meant that the apoptotic induction mechanism of Cos on HCC cells may be different from VOSL, C+2D and Dehy (Figure 3D).

### VOSL inhibits the capbility of HCC cell motility

**Figure 4:**
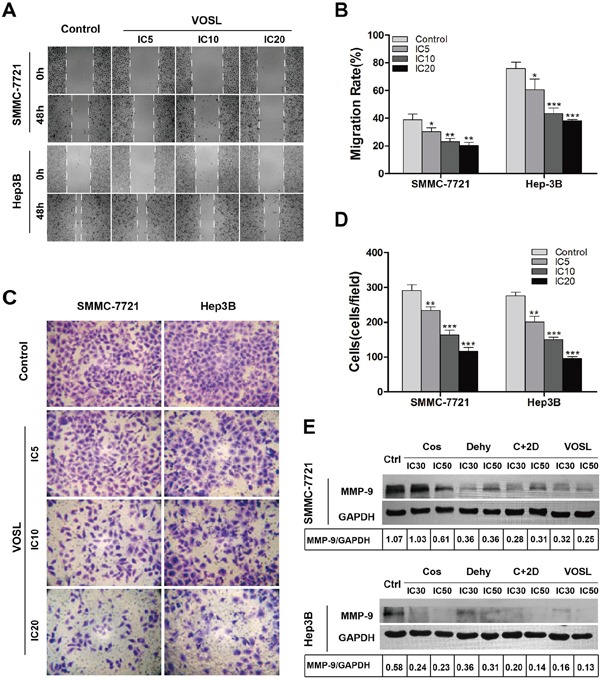
VOSL inhibits the motility activity of HCC cells **A** and **B.** HCC cells were treated with VOSL (IC5, IC10, IC20) for 48 h, and the effect of VOSL on cell migration was measured by scarification test. The migration rates of HCC cells were presented as mean ± SD; *p < 0.05, **p < 0.01 and ***p < 0.001 compared with the control group. **C** and **D.** After incubation with VOSL at IC5, IC10 and IC20 for 48 h, the invasive property of HCC cells was tested in Transwell plates; original magnification 200×. The invasive cells were presented as mean ± SD; **p < 0.01 and ***p < 0.001 compared with the control group. **E.** HCC cells were treated with Cos, Dehy, C+2D or VOSL (IC30, IC50) for 48 h, and then the expression level of MMP-9 in HCC cells was examined by Western blot.

The majority of HCC patients die of tumor recurrence and metastasis, therefore, inhibiting the capbility of HCC cell motility is an attractive strategy for its treatment [10]. We examined the possible effects of VOSL, C+2D, Cos, and Dehy on HCC cell migration and invasion through wound healing and Transwell assay. The results indicated that VOSL, C+2D, Cos, and Dehy, at low concentrations that did not notably reduce the viability of HCC cells, dose-dependently reduced the capabilities of HCC cell migration and invasion when compared with the untreated group (Figure 4A-4D; Supplementary Figure S3 and S4). Moreover, MMP-9, a major member of matrix metalloproteinases (MMPs), was measured through Western blot, the results showed that its expression was significantly reduced in both SMMC-7721 and Hep3B cells after treatment (Figure 4E).

### VOSL inhibits EGFR activation in HCC cells

**Figure 5:**
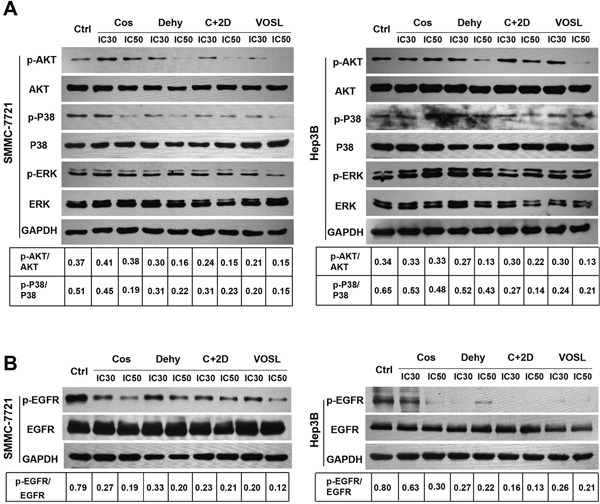
VOSL suppressed the EGFR/MEK/P38 and EGFR/PI3-K/Akt pathways in HCC cells **A** and **B.** HCC cells were treated with Cos, Dehy, C+2D or VOSL at concentrations of 0, IC30 and IC50 for 48h, and then the expression levels of the relevant factors (P38, p-P38, Akt, p-Akt, ERK, p-ERK, EGFR and p-EGFR) were determined by Western blot.

Activation of the extracellular signal-regulated kinases (ERK), the Jun N-terminal kinases (JNK) and the p38 MAPKs plays an important part in regulating of cancer cell biological characteristics [14, 15]. PI3-K/Akt signaling is widely involved in controlling the proliferation, survival or death of cancer cells [16]. Therefore, the effects of VOSL, C+2D, Cos, and Dehy on MAPK and Akt activation were examined. MEK/P38 cascade activation in SMMC-7721 and Hep3B cells was dose-dependently suppressed by VOSL, C+2D, Cos, and Dehy, but they had no effect on the activation of ERK. Meanwhile, VOSL, C+2D, and Dehy dose-dependently decreased p-Akt levels in SMMC-7721 and Hep3B cells, however, the p-Akt level had no obvious changes in the Cos-treated group (Figure 5A).

Since MEK/P38 and PI3-K/Akt is the primary downstream cascades of receptor tyrosine kinases (RTKs), meanwhile, EGFR is the most important RTK in cancer cells [17, 18]. Therefore, we speculated that VOSL may act as an EGFR inhibitor to suppress EGFR activation. In order to validate this speculation, we determined the EGFR activation in the control group and the treatment groups. The results revealed that the phosphorylated EGFR (p-EGFR) levels in HCC cells were significantly suppressed after VOSL, C+2D, Cos, and Dehy treatments (Figure 5B).

### VOSL suppresses RTKs-mediated signaling in HCC xenografts

**Figure 6:**
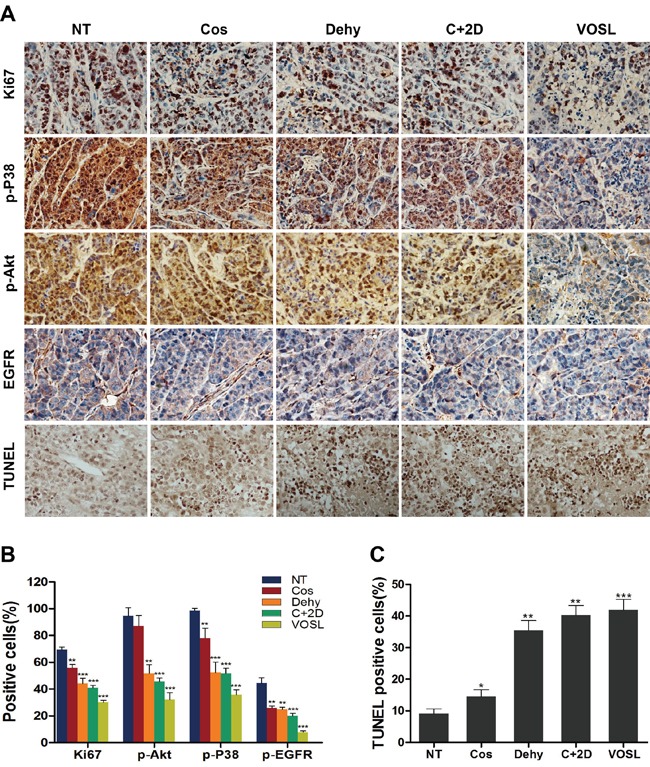
Expressions of the factors in SMMC-7721 xenografts after treatments **A.** The expression of Ki-67, p-P38, p-Akt, p-EGFR and the apoptotic cells in xenografts were examined by immunohistochemistry and TUNEL assay; original magnification 200×. **B** and **C.** The positive cells of the relevant factors and the apoptotic cells in xenografts were presented as mean ± SD; *p < 0.05, **p < 0.01 and ***p < 0.001 compared with the NT control group.

The expression of relevant factors and the number of apoptotic cells in xenograft tumors were detected by immunohistochemistry and TUNEL assay, respectively. VOSL, C+2D, Cos, and Dehy treatments could induce SMMC-7721 cell apoptosis in xenograft tumors, among them, VOSL exerted the greatest effect on inducing cell apoptosis (Figure 6A and 6C). The phosphorylation of Akt was also blocked by VOSL, C+2D, and Dehy treatments in the SMMC-7721 xenografts, except the Cos-treated group (Figure 6A and 6B). Simultaneously, the expression of Ki67, p-P38 and p-EGFR in all treatment groups was reduced compared with the control group, and their lowest expression levels were observed in the VOSL-treated group (Figure 6A and 6B). Therefore, we concluded that VOSL may inhibit HCC progression through suppressing the RTKs-mediated signaling pathways.

## DISCUSSION

HCC is one of the common malignances and has high mortality due to frequent recurrence, metastasis and multi-drug resistance [19, 20]. Our study demonstrated that VOSL, C+2D, Cos and Dehy, acting as EGFR inhibitors, exhibited anti-HCC activity, and VOSL showed the best anti-HCC activity among the tested groups.

Unlimited proliferation is a hallmark of cancer cells, thus proliferation inhibition and apoptosis induction are the two main mechanisms of chemotherapeutic agents [21]. In this study, VOSL, C+2D, Cos and Dehy markedly inhibited the proliferation of SMMC-7721 and Hep3B cells, interfered the progression of cell cycle, and also triggered apoptosis to inhibit the progress of HCC. The apoptotic mechanisms in HCC cells induced by Cos may be partially different from those induced by VOSL, C+2D or Dehy due to the ratio of cleaved-Caspase3 to Caspase3 was obviously increased in the VOSL, C+2D or Dehy-treated groups, but it was no obvious change in the Cos-treated group. Activation of Caspase 3 is always involved in the regulation of intrinsic and extrinsic apoptotic pathways, and the activity of P38 has great relationship with the endoplasmic reticulum (ER) stress apoptotic pathway [10]. In this study, we also found that the phosphorylation of P38 in SMMC-7721 and Hep3B cells was dose-dependently inhibited by VOSL, C+2D, Cos, and Dehy. Therefore, we suggested that VOSL, C+2D or Dehy-induced HCC cell apoptosis may adopts at least two different apoptotic pathways, but Cos-induced HCC cell apoptosis possibly employs the ER stress pathway.

Numerous studies indicated that Cos and Dehy can modulate cell cycle progression by activating the P21/P27 pathway, which lead to cell cycle arrest in S or/and G2/M phases [22, 23]. In this study, we indeed observed that VOSL, C+2D, Dehy and Cos induce HCC cell cycle arrest in S or/and G2/M phases. MMP-9 can degrade some extracellular matrixs, and the change of MMP-9 expression is closely related to tumor metastasis and invasion in various tumors [24–28]. Our results from Scarification and Transwell assays indicated that VOSL, C+2D, Cos, and Dehy at low concentrations significantly reduced the capabilities of HCC cell migration and invasion. In addition, we observed the down-regulation of MMP-9 level in the VOSL, C+2D, Cos or Dehy- treated HCC cells.

Accumulating evidence showed that MAPK activation play a vital role in HCC development [17]. We found that VOSL, C+2D, Cos and Dehy treatments significantly depressed MEK/P38 cascade activation, but didn't influence ERK activation. PI3-K/Akt pathway is also involved in HCC progression. The activated Akt has been considered as an indicator for HCC recurrence and poor prognosis of patients [29]. Our results revealed that VOSL, C+2D, Cos and Dehy significantly suppressed the PI3-K/Akt signaling in HCC cells. Taken together, the simultaneous suppression of MEK/P38 and PI3-K/Akt signaling always by VOSL represents an attractive strategy for treating HCC.

RTKs are glycoproteins spanning cell membrane, which interact with growth factors and in turn cause the activation of intracellular catalytic domains, whereby transmit cellular signals [30]. EGFR is the most important RTK in cancer cells and overexpresses in a variety of malignancies [31, 32]. It can activate distinct downstream kinases, and leads to the phosphorylation of MAPK kinases and Akt [33–36]. Considering VOSL could simultaneously inhibit MEK/P38 and PI3-K/Akt signaling, we thought that VOSL may influence EGFR activation in HCC cells. Interestingly, our data showed that VOSL, C+2D, Cos and Dehy significantly suppressed the phosphorylation of EGFR in SMMC-7721 and Hep3B cells, which confirms our conjecture.

**Figure 7:**
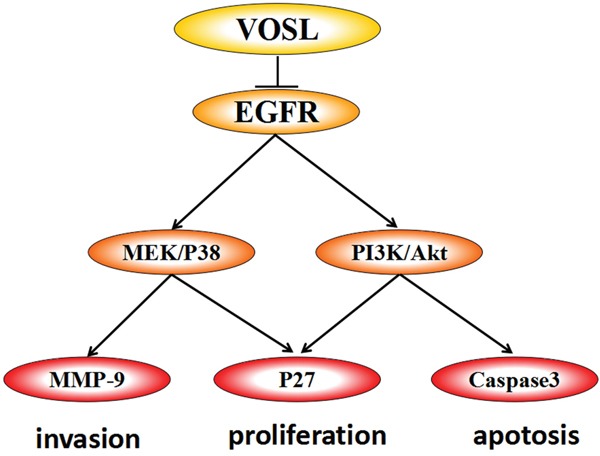
The model of how VOSL exerts antitumor efficacy in SMMC-7721 and Hep3B cells VOSL exerts antitumor efficacy by inhibiting EGFR signaling pathway in hepatocellular carcinoma. The correlative pathways PI3K/Akt and MEK/P38 were suppressed, which resulted in downregulation of MMP-9 and Caspase3.

Although both Cos and Dehy are natural sesquiterpene lactones, their anti-HCC molecular mechanisms seem to be different. Combination treatment of these two compounds (C+2D) showed a synergistic anti-HCC effect in the SMMC-7721 xenografts. In addition, we speculated that there should be indirect anti-HCC compounds besides direct anti-HCC compounds, Cos and Dehy, in VOSL because VOSL exhibits better anti-HCC effect than C+2D *in vivo*. Therefore, VOSL exhibited the therapy characteristics of multiple-components and multiple-targets, which were Cos and Dehy acted on HCC xenografts directly, and other active ingredients in VOSL may act on nude mice to exert indirectly anti-HCC activity. Figuring out the indirect anti-HCC ingredients in VOSL and exploring their associated mechanism will be our topic of further research. In conclusion, our experiments showed that VOSL suppressed HCC progression by regulating EGFR pathway (Figure 7), which could be a useful drug candidate in HCC therapy.

## MATERIALS AND METHODS

### Compounds

VOSL was prepared as previously described [9]. Briefly, ten g of *Saussurea lappa* roots were crushed into powder, and extracted with 100 mL of hexane for 1h, The filtrates were evaporated to get VOSL, and then were analyzed by ultraperformance liquid chromatography coupled to quadrupole-time-of-flight mass spectrometer (Waters, Milford, MA, USA). Commercial Cos and Dehy were purchased from Shanghai Yuanye Biotech CO., Ltd and its purity is more than 98%. VOSL, Cos and Dehy were dissolved in dimethyl sulfoxide to 10 mg/mL, and then diluted to appropriate concentrations with cell culture medium.

### Cell culture

Human HCC cell lines, SMMC-7721, Hep3B, HepG2, Huh-7, HCC-LM3, and two normal hepatic cell lines, WRL-68 and L02, were cultured in DMEM with 10% fetal bovine serum (HyClone, Logan, UT, USA), and incubated at 37°C, 5% CO_2_.

### Cell proliferation

The ratio of Cos to Dehy is about 1 to 2 by weight in VOSL, therefore, in the following experiments, we generally designed four tested groups, the Cos group, Dehy group, C+2D (Cos/Dehy=1:2, w/w; simulating the composition ratio of VOSL) group, and VOSL group, to treat HCC cells. Cell viability was detected by MTT assay. 0, 5, 10, 15, 20 μg/ml of the tested samples (Cos, Dehy, C+2D and VOSL) were added in cultured cells. After 48 h incubation, MTT reagent was added to each well, then the absorbanc at 492 nm was detected and the IC50 value of every tested sample was calculated using the Trimmed Spearman-Karber Method [13].

### Cell clonogenic and cell cycle assays

To determine long-term effects of Cos, Dehy, C+2D or VOSL on the proliferation of SMMC-7721 and Hep3B cells, cells were exposed to the tested samples at IC10, IC30 and IC50 concentrations for 48 h, respectively, and rinsed with fresh medium. The resulting adherent cells were cultured continuously for 14 days in normal culture condition. Finally, the cells were fixed and stained with crystal violet for counting the clone formation.

The distribution of cell cycle was detected by propidium iodid (PI) staining. SMMC-7721 and Hep3B cells were treated with Cos, Dehy, C+2D or VOSL at various concentrations (IC10, IC30 and IC50) for 48 h. The harvested cells were fixed in alcohol (70%) for 24 h at 4°C, stained with PI for 30 min, then analyzed with flow cytometry (Becton Dickinson, Franklin Lakes, NJ, USA).

### Apoptosis analysis

Hoechst was used to determine the morphological change of apoptotic cells. SMMC-7721 and Hep3B cells were treated with Cos, Dehy, C+2D or VOSL at IC50 concentration for 48 h, and then stained with Hochest 33342 (Beyotime Biotechnology, Shanghai, China) for 15 min. A fluorescence microscope was used to observe the morphological change of apoptotic cells.

SMMC-7721 and Hep3B cells were treated with Cos, Dehy, C+2D or VOSL at IC10, IC30 and IC50 concentrations, respectively, for 48 h, and stained with Annexin V-FITC/PI (Yesen, Biotechnology, Shanghai, China) for 10 min, and then analyzed by FACS Calibur (Becton Dickinson, Franklin Lakes, NJ, USA) to calculated the apoptotic ratio of cells.

### Migration and invasion activity assays

Scarification test was used to evaluate the migration of HCC cells. SMMC-7721 and Hep3B cells were planted in 6-well plates and wounds were created with a micro-pipette tip in the cell monolayer. The cells were exposed to Cos, Dehy, C+2D or VOSL at IC5, IC10, and IC20 concentrations, respectively, for 48 h, and their migration distances were measured by Photoshop.

Transwell assay with extracellular matrix (ECM) was performed to evaluate the influence of Cos, Dehy, C+2D and VOSL on tumor invasion. SMMC-7721 and Hep3B were exposed to Cos, Dehy, C+2D or VOSL at IC5, IC10, and IC20 concentrations for 48 h, and the infiltrated cells were stained with crystal violet and statistically analyzed by microscope.

### Protein extraction and western blot analysis

SMMC-7721 and Hep3B cells were cultured and incubated with Cos, Dehy, C+2D or VOSL at IC30 and IC50 concentrations for 48 h, respectively. The harvested cells were prepared to extract total protein. The extracted protein was separated on 8% - 12% SDS-PAGE, then transferred onto PVDF membrane (Beyotime Institute of Biotechnology). The blot was blocked with non-fat milk, incubated with the primary antibodies (Caspase3, cleaved-Caspase3, Akt, p-Akt, P38, p-P38, ERK, p-ERK, EGFR and p-EGFR) and then with the secondary antibodies. Signals were developed using ECL reagents (GE Healthcare Life Sciences, Shanghai, China). GAPDH was used as an inner control.

### *In vivo* tumor xenograft study

All procedures involving animals were approved by the Experimental Animal Ethics and Management Committee. Male nude mice (4 weeks old; BALB/c-nude) were purchased from SLAC Laboratory Animal Center of Chinese Academy of Sciences (Shanghai, China). SMMC-7721 cells were injected subcutaneously into the axillary fossa of nude mice (4×10^6^ cells in 150 μl PBS). Tumors were developed at 10 days after cell injection. The treatment was started when tumors reached a size of 2 - 3 mm in diameter. Thirty-six mice were randomly divided into six groups. The Cos, Dehy, C+2D and VOSL-treated groups were injected intraperitoneally at a dose of 15 mg/kg/day, respectively. The positive and negative control group were administrated with 5-Fluorouracil (5-Fu) at a dose of 15 mg/kg/day, and an equal volume of vehicle, respectively. Tumor size was monitored every three days, and tumor volume was calculated using the formula: tumor volume=0.5×*a*×*b*^2^, where *a* and *b* represent the largest and smaller diameters, respectively.

Tumor-bearing mice were sacrificed after 24 times of administrations. Tumors were collected and weighed, and then prepared the consecutive sections for examining the expression of p-AKT, p-P38, Ki-67 and p-EGFR by immunohistochemistry. The apoptotic cells in xenograft tumors were detected by TUNEL (Beyotime Institute of Biotechnology, Shanghai, China). The positive cells were observed under microscope within 5 medium-power fields at magnification 200×.

### Statistical analysis

The *in vitro* experimental data were derived from at least three independent experiments, and the *in vivo* data were collected from 6 mice. All data were expressed as mean±SD, and the significant difference was determined by using the Student's *t*-test. The statistical significance was set at *p* < 0.05.

## SUPPLEMENTARY FIGURES


