# Supplementary material for: Volatile oil from Saussurea lappa exerts antitumor efficacy by inhibiting epithelial growth factor receptor tyrosine kinase-mediated signaling pathway in hepatocellular carcinoma
Source: Oncotarget. 2016 Oct 28;7(48):79761–73. doi: 10.18632/oncotarget.12962 (PMC5346749; doi:10.18632/oncotarget.12962)
Supplement: Supplementary file 1 [file oncotarget-07-79761-s001.pdf]

Volatile oil from *Saussurea lappa* exerts antitumor efficacy by inhibiting epithelial growth factor receptor tyrosine kinase-mediated signaling pathway in hepatocellular carcinoma

SUPPLEMENTARY FIGURES

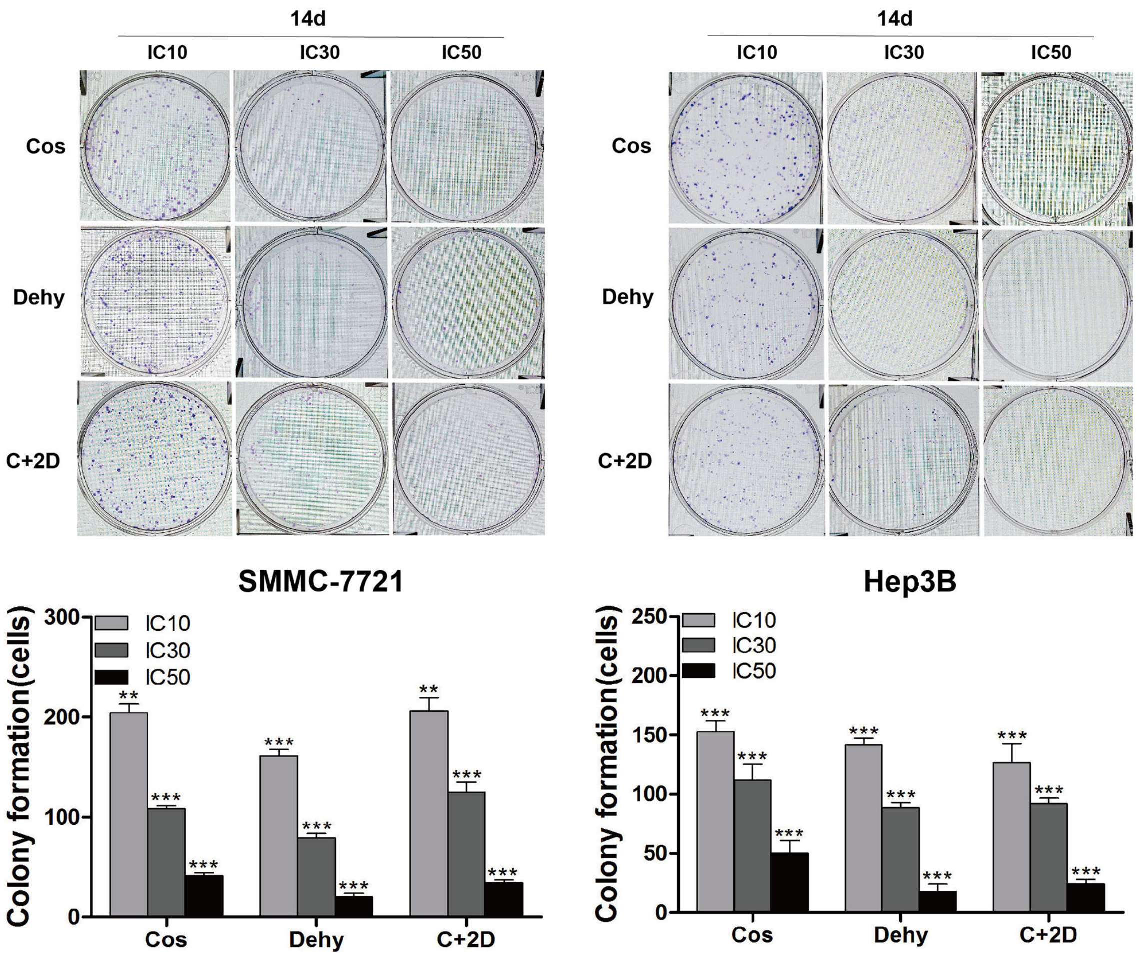

Supplementary Figure S1: Colony formation assay was used to confirm the long-term effect of Cos, Dehy and C+2D at different concentrations (IC10, IC30, IC50) for 48 h, and the colony formation was presented as mean ± SD; **p < 0.01 and ***p < 0.001 compared with the control group.

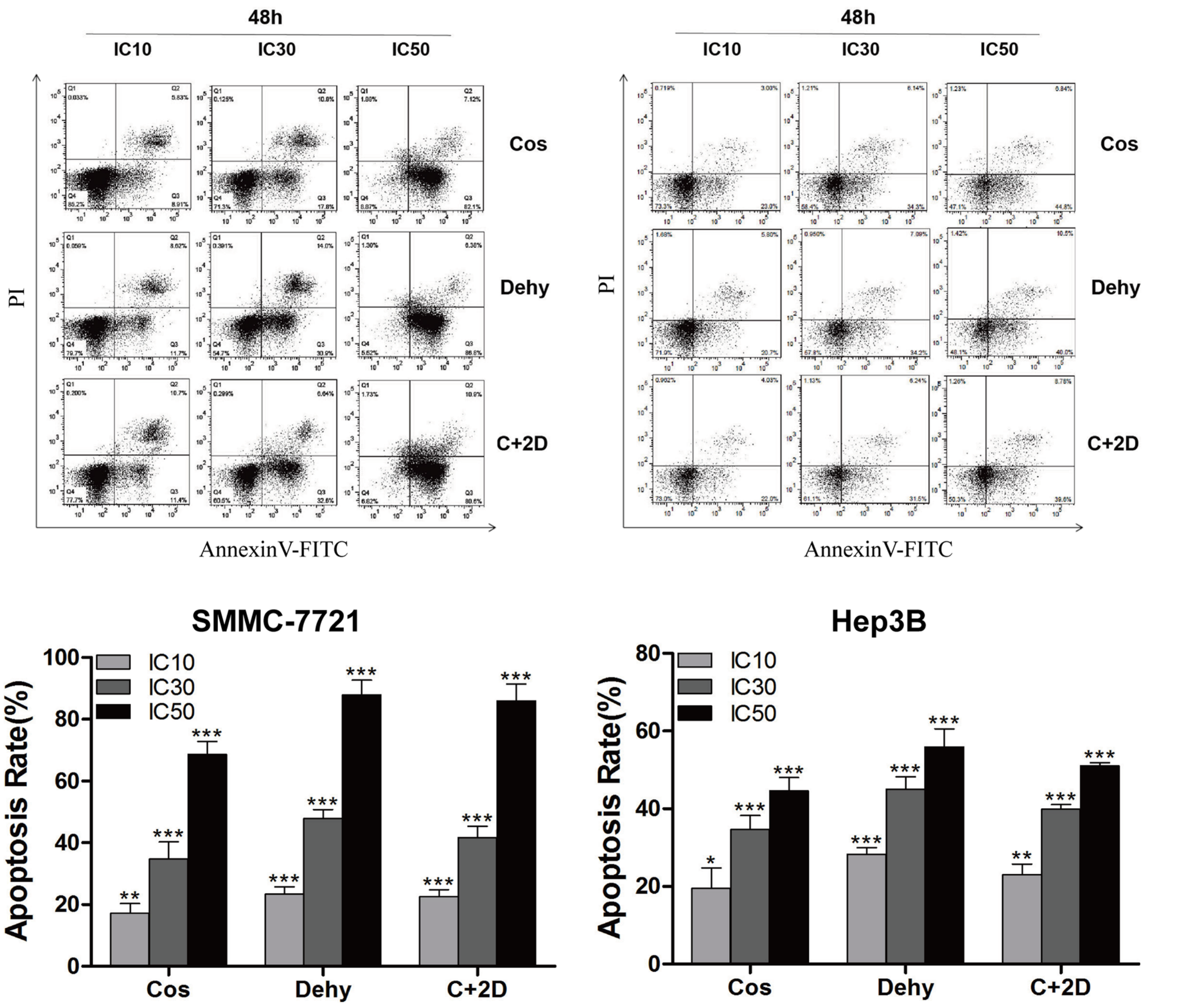

Supplementary Figure S2: HCC cells were treated with Cos, Dehy, C+2D (IC10, IC30, IC50) for 48 h and the apoptotic cells were determined by flow cytometry. The apoptotic percentages were presented as mean ± SD; *$p < 0.05$, **$p < 0.01$ and ***$p < 0.001$ compared with the control group.

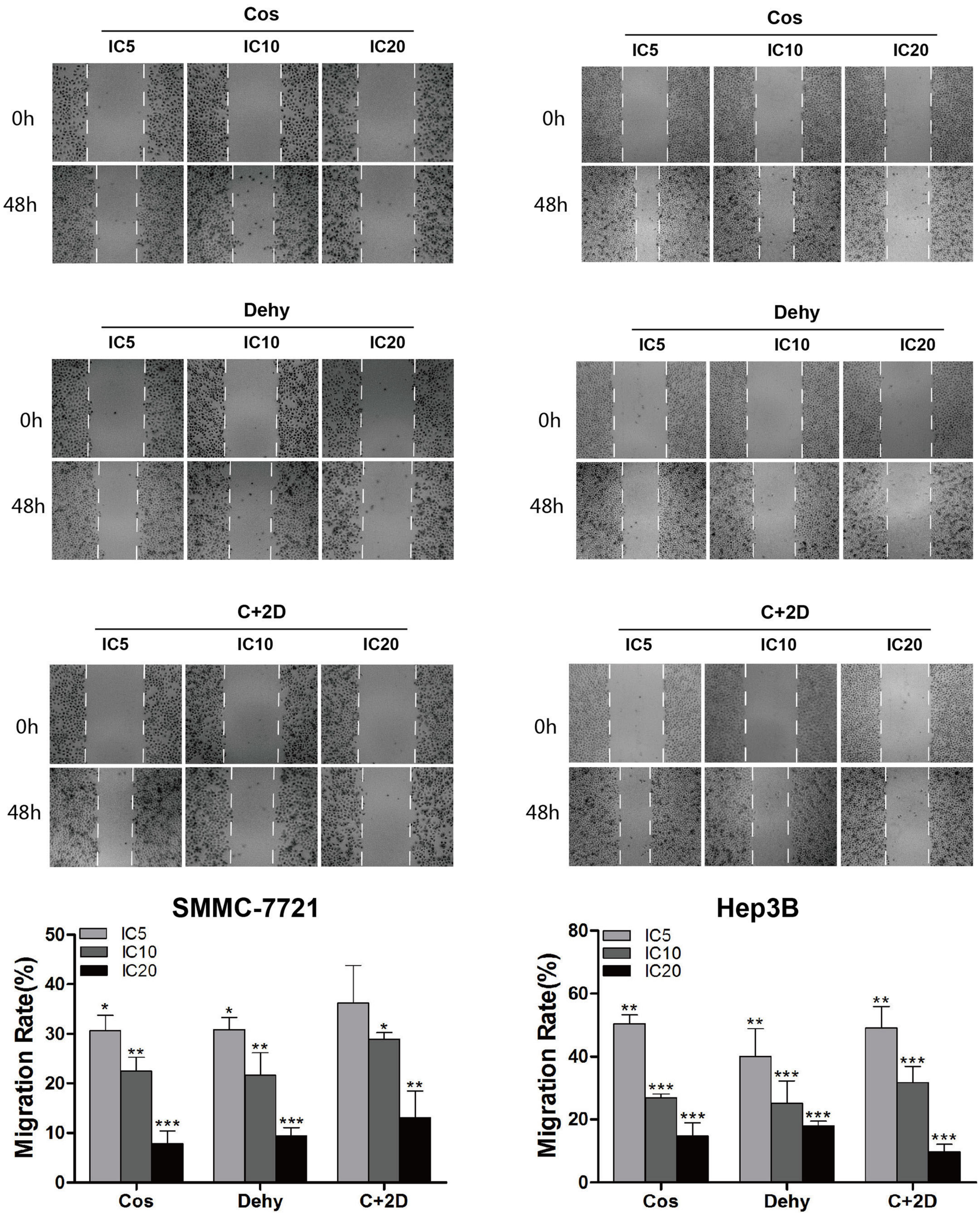

Supplementary Figure S3: HCC cells were treated with Cos, Dehy and C+2D (IC5, IC10, IC20) for 48 h, and the effect on cell migration was measured by scarification test. The migration rates of HCC cells was presented as mean ± SD; $*p < 0.05$, $**p < 0.01$ and $***p < 0.001$ compared with the control group.

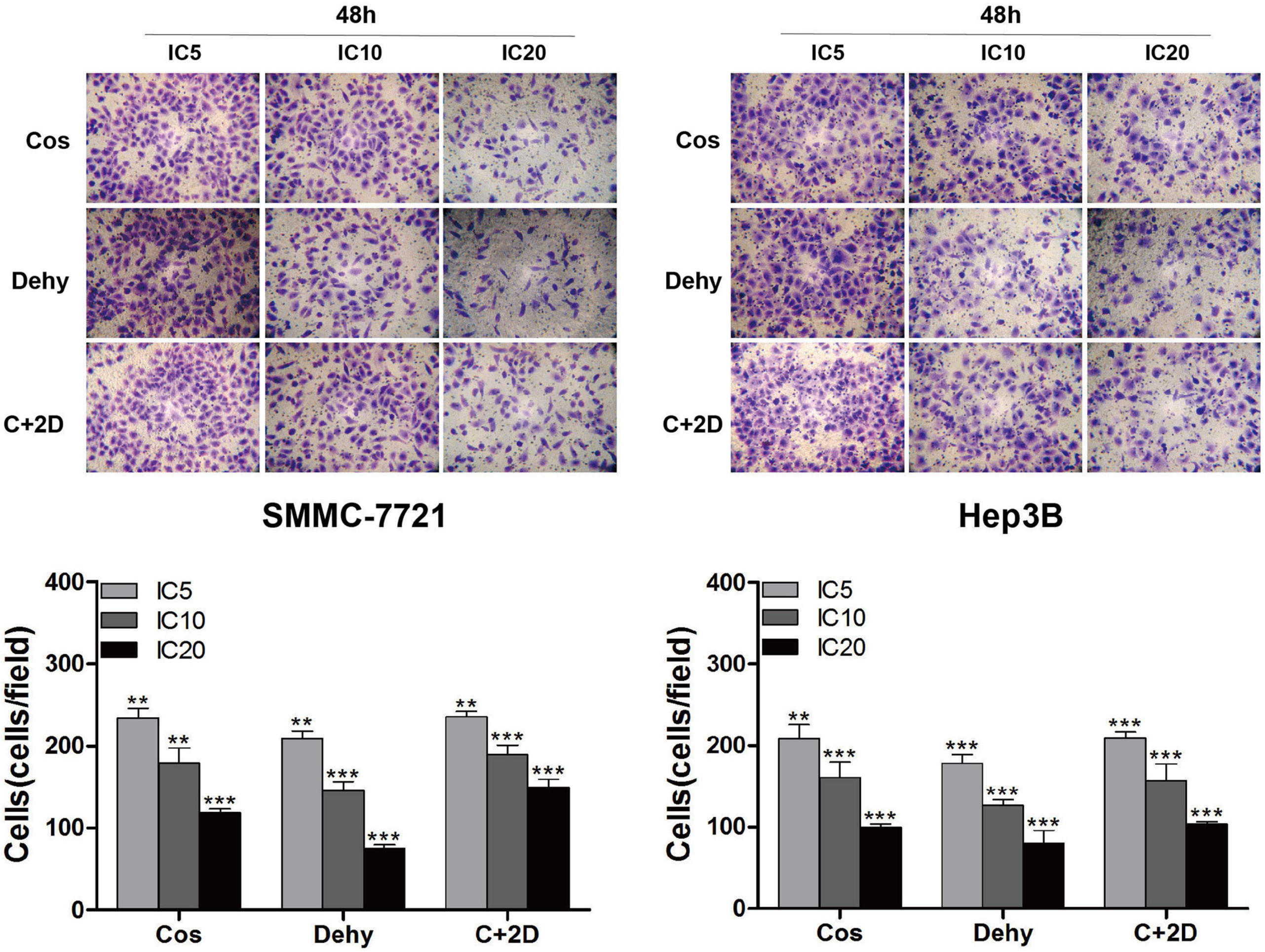

Supplementary Figure S4: After incubation with Cos, Dehy and C+2D at IC5, IC10 and IC20 for 48 h, the invasive property of HCC cells was tested in Transwell plates; original magnification 200×. The number of invasive cells was presented as mean ± SD; $^{**}p < 0.01$ and $^{***}p < 0.001$ compared with the control group.
